# Three-Locus Sequence Identification and Differential Tebuconazole Sensitivity Suggest Novel *Fusarium equiseti* Haplotype from Trinidad

**DOI:** 10.3390/pathogens9030175

**Published:** 2020-03-01

**Authors:** Ria T. Villafana, Sephra N. Rampersad

**Affiliations:** Department of Life Sciences, Faculty of Science and Technology, The University of the West Indies, St. Augustine, Trinidad and Tobago; riatvill@hotmail.com

**Keywords:** azoles, DMI, *Fusarium incarnatum-equiseti* species complex

## Abstract

The *Fusarium* incarnatum-equiseti species complex (FIESC) consists of 33 phylogenetic species according to multi-locus sequence typing (MLST) and Genealogical Concordance Phylogenetic Species Recognition (GCPSR). A multi-locus dataset consisting of nucleotide sequences of the translation elongation factor (*EF-1*α), calmodulin (*CAM*), partial RNA polymerase largest subunit (*RPB1*), and partial RNA polymerase second largest subunit (*RPB2*), was generated to distinguish among phylogenetic species within the FIESC isolates infecting bell pepper in Trinidad. Three phylogenetic species belonged to the Incarnatum clade (FIESC-15, FIESC-16, and FIESC-26), and one species belonged to the Equiseti clade (FIESC-14). Specific MLST types were sensitive to 10 µg/mL of tebuconazole fungicide as a discriminatory dose. The EC50 values were significantly different among the four MLST groups, which were separated into two homogeneous groups: FIESC-26a and FIESC-14a, demonstrating the “sensitive” azole phenotype and FIESC-15a and FIESC-16a as the “less sensitive” azole phenotype. CYP51C sequences of the Trinidad isolates, although under positive selection, were without any signatures of recombination, were highly conserved, and were not correlated with these azole phenotypes. CYP51C sequences were unable to resolve the FIESC isolates as phylogenetic inference indicated polytomic branching for these sequences. This data is important to different research communities, including those studying *Fusarium* phytopathology, mycotoxins, and public health impacts.

## 1. Introduction

Bell pepper (*Capsicum annuum* L.) is one of the most widely cultivated vegetable crops in the world. Over the last decade, the world’s production and consumption of bell peppers have been steadily increasing. More than 70% of the world’s bell peppers are produced in Asia, with China being the largest producer of bell peppers [1]. The *Fusarium* disease of bell pepper, resulting in external fruit rot, is caused by *F. oxysporum*, *F. proliferatum*, *F. solani*, *F. lactis*, and *F. incarnatum*-*equiseti* species complexes and has been reported to occur in Belgium, Canada, the Netherlands, and the United Kingdom [2,3,4,5]. Symptoms of external infection include water-soaked, sunken lesions that expand to or originate from the calyx end of the fruit—either in the ripe or immature green stage. Internal fruit rot can also develop where the seeds and placenta become infected and turn black with rot. Residue management, crop rotation, seed treatments, and fungicide use form the general integrated disease management strategies for controlling fungal diseases of bell pepper, and while there is no seed treatment or fungicide that will eliminate these diseases entirely, certain fungicides have been reported to reduce inoculum load (https://www.cropscience.bayer.ca). There are no currently available bell pepper cultivars that are resistant or tolerant to *Fusarium* disease but yield and resistance remain the paramount breeding targets for sustainable production.

Between 2010 and 2014, a survey of the major bell pepper growing areas in Trinidad was carried out from which it was reported that fruit rot of bell peppers was caused by two fungal pathogens, *Colletotrichum truncatum* (synonym *C. capsici*; [6]) and *Fusarium* species including members of the *Fusarium incarnatum-equiseti* species complex (FIESC) [7,8]. FIESC isolates are pervasive soil inhabitants and are pathogenic to a range of economically important plant species, including cereals, fruits, and vegetables [8,9,10,11]. Members of this species complex are able to produce several mycotoxins, which upon consumption, pose health risks to animals and humans [10,12,13]. In humans, several species and species complexes are associated with fusariosis, including *F. incarnatum-equiseti* [14,15,16,17].

The *EF-1*α (translation elongation factor-1α) gene sequence, with a sequence similarity threshold of 99.4%, is a suitable genetic marker for discriminating among *Fusarium* spp. and allows for discrimination of genotypes to the intraspecific level [18,19]. However, reliable identification of unknown isolates and phylogenetic inference is based on sequence data that must be generated for multiple loci apart from *EF-1*α and includes RNA polymerase largest and second-largest subunits (*RPB1* and *RPB2*), and Calmodulin (*CAM*), using the same sequence similarity threshold as for *EF-1*α [19]. O’Donnell et al. [14] concluded that highly divergent β-tubulin paralogs existed in the genomes of FIESC, which excludes the use of this locus for FIESC phylogenetic inference. The high similarity of ITS (internally transcribed spacer region, ITS1-5.8S-ITS2) sequences (>98%) also disqualifies this marker for phylogenetic demarcation of members of this species complex. The identification of FIESC isolates in Trinidad for the 2010-2014 survey was based on *EF-1*α and ITS sequence comparisons in the *FUSARIUM*-ID database [8]. There is a lack of Latin binomials for most of the species within the FIESC and presents systematic challenges in terms of taxonomic demarcation for this complex, which was initially based on homoplastic morphological characters and ITS sequence comparisons. Therefore, the present study sought to confirm and expand the identities of the *Fusarium* isolates infecting bell pepper in Trinidad in a re-sampling effort using a three-locus MLST scheme and up-to-date phylogenetic species identities provided by *Fusarium* MLST (http://www.wi.knaw.nl/Fusarium/). Accurate species assignment is important for epidemiological studies and guiding disease management.

Chemical control using methyl benzimidazole carbamates (MBCs) [FRAC code: 1] is the main approach to disease management in bell peppers in Trinidad. MBCs function to disrupt β-tubulin monomerization, which, in turn, affects microtubule arrangement and mitotic spindle formation [20]. Ramdial et al. [21] indicated that, in Trinidad, resistance to MBCs was detected in the *C. truncatum* population infecting bell pepper fruit and that FIESC isolates had significantly lower EC50 values compared to *C. truncatum*. However, it was unclear whether these EC50 values for FIESC isolates reflected resistance or dosage requirements for this fungicide. FRAC [22] warns of a high risk of resistance, resulting from the over-use of benzimidazoles and resistance management is an important consideration for controlling diseases caused by *Fusarium* species, e.g., *F. graminearum*, *F. oxysporum* f. sp. *gladioli* and *F. oxysporum* f. sp. *lilli* [23,24,25,26]. Alternatives to benzimidazole fungicides to control fungal diseases in bell pepper in Trinidad are necessary to reduce the risk of MBC fungicide resistance in the FIESC pathogen population. Recent research on the differences in antifungal susceptibility between species and isolates also demonstrates the need for correct species-level identification [15].

Azoles are the largest, most commercially successful class of sterol 14α-demethylation inhibiting (DMI) fungicides [FRAC code: 3], and there is a wide variation in the activity spectra of different DMI fungicides [22,27]. Sterol 14α-demethylase is required for sterol biosynthesis in fungi and is also the target of azole compounds that inhibit ergosterol biosynthesis in fungi, which halts fungal growth as a result of dysregulation of fluidity, permeability, and rigidity of fungal plasma membranes [28,29,30,31,32,33]. Rapid market growth for triazole fungicides has been reported for North America, Europe, and the Asia Pacific, while in the UK, the Netherlands, and Denmark, prothioconazole, epoxiconazole, and tebuconazole are the main azole fungicides used in crop production [34]. Although tebuconazole consists of a pair of enantiomers that results in apparent enantioselective fungicidal activity, uptake and translocation [35,36], it is among the most commonly used fungicides to control *F. graminearum* and *Fusarium* head blight diseases of wheat in many countries [37,38]. 

Azole fungicides inhibit sterol substrate binding and, therefore, function to inhibit cytochrome P450 sterol 14 α-demethylase CYP51 enzyme activity. CYP51 genes encode sterol 14α-demethylases, which appear to retain strict catalytic function in the oxidative removal of the 14α-methyl group from sterol precursors across all phyla [39]. Three CYP51 paralogues have been described for *Fusarium* species, of which CYP51C is unique to this genus [40]. In a study of *F. graminearum* isolates by Fan et al. [41], *Fg*CYP51C served as a virulence factor and can indirectly affect sterol 14α-demethylation even though it no longer functions as a sterol 14α-demethylase and where deletion of *Fg*CYP51C, either as single or double mutants (*ΔFg*CYP51AC), had no effect on azole sensitivity, which suggested that CYP51C is a neo-functionalized paralogue. Conversely, in a separate study by Liu et al. [42] also of *F. graminearum* isolates, there was increased sensitivity to tebuconazole and prochloraz in *ΔFg*CYP51C mutants. *Fusarium* can engage a number of other virulence factors that function as specialized genes or as part of complex pathways [43,44,45,46,47,48,49]. The paucity of information regarding evolutionary maintenance of this CYP51C paralogue and conflicting evidence concerning its involvement in azole resistance indicates that these relationships should be examined for the following reasons: (i) azoles are among the main fungicides used in crop production worldwide, (ii) cross-resistance between agricultural and clinical azoles impact on opportunistic fusaria that cause diseases in humans and animals, and (iii) alternative approaches to avoid or delay azole resistance may include the inhibition of CYP51 with substrate analogs, however, without data on the genetic structure of CYP51C in relation to azole resistance or tolerance, this strategy remains incomplete.

The main objectives of this study were, therefore, to (i) identify members of FIESC to the phylogenetic species level and clarify the phylogenetic relationships among FIESC sequences from Trinidad and other geographical regions using a three-locus sequence comparison approach, (ii) determine the sensitivity of Trinidad FIESC isolates to tebuconazole based on in vitro bioassays, and (iii) examine CYP51C genetic structure for correlation with azole fungicide sensitivity. This data is important to different research communities, including those studying *Fusarium* phytopathology, mycotoxins, and public health impacts.

## 2. Results

### 2.1. The Identification and Phylogenetic Placement of Isolates

**Figure 1:**
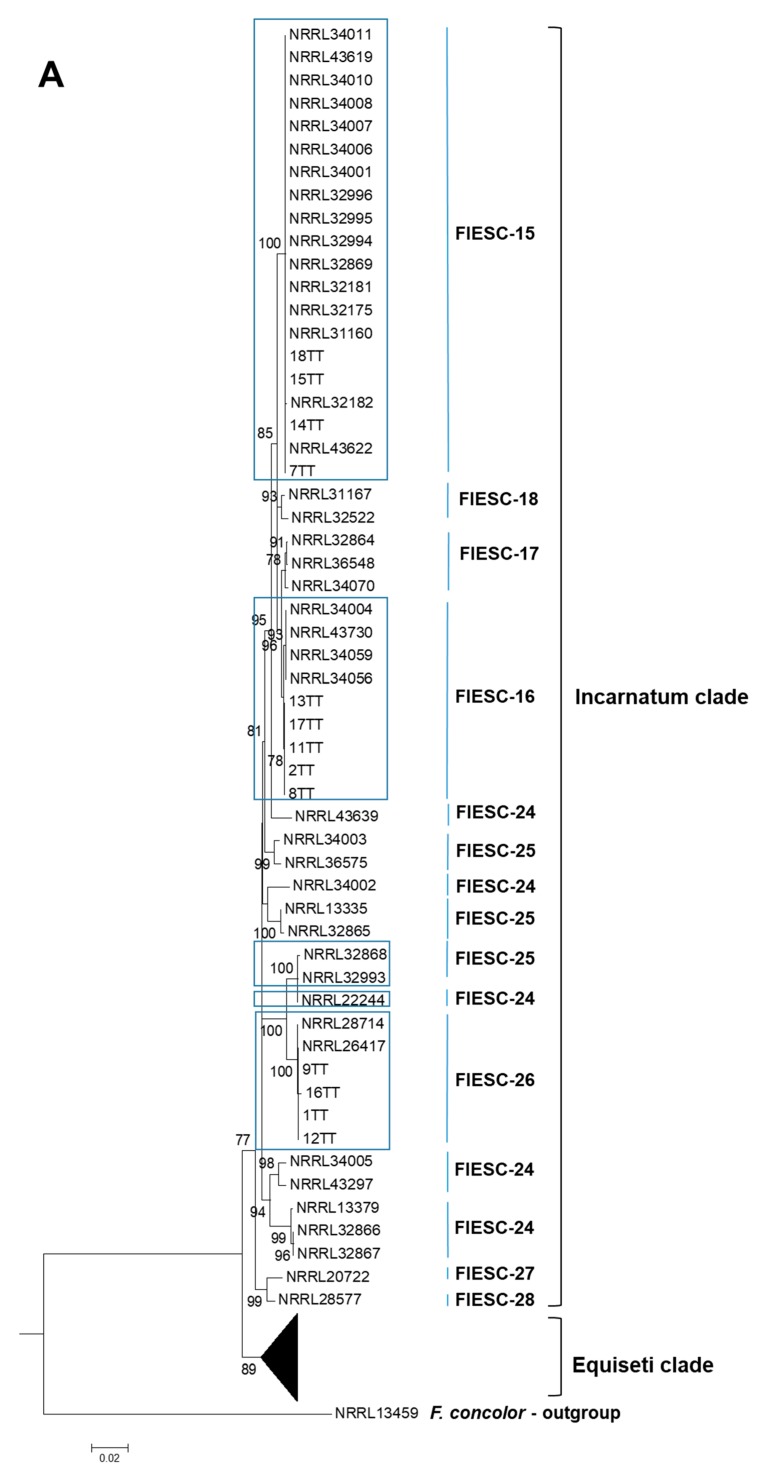

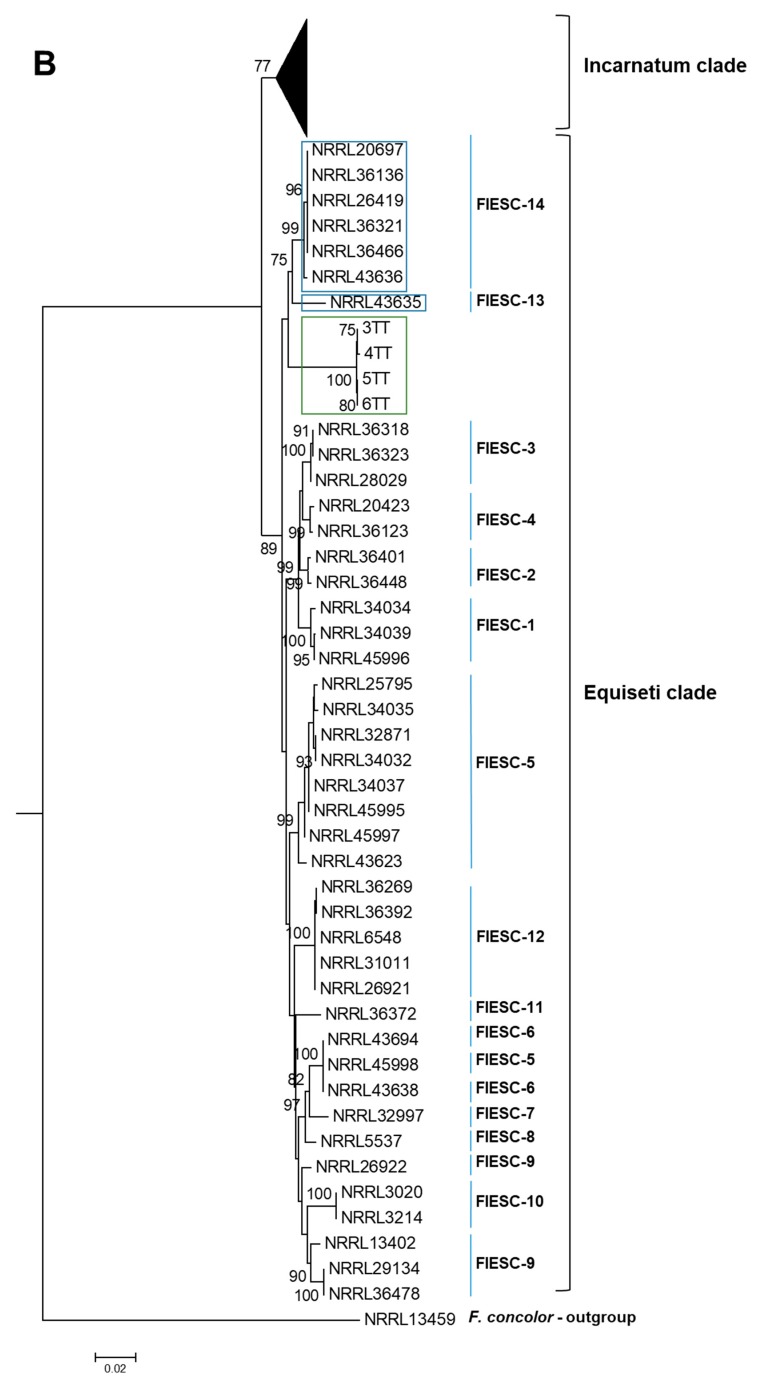
(**A**,**B**). Phylogenetic analysis of FIESC sequences based on concatenated partial nucleotide sequences of *EF-1α*, *RPB2*, and *CAM* genes. The phylogenetic relationships were inferred by using the Maximum Likelihood method based on the General Time Reversible model, as the best fit model, with 1000 bootstrapped replicates [50]. The tree with the highest log-likelihood is shown. The percentage of trees in which the associated taxa clustered together is shown next to the branches. The tree is drawn to scale, with branch lengths measured in the number of substitutions per site. The analysis involved 103 nucleotide sequences. All positions containing gaps and missing data were eliminated. There were a total of 1804 positions in the final dataset. Evolutionary analyses were conducted in MEGA6 [51]. Black triangle depicts a collapsed branch; blue boxes indicate sequences belonging to a confirmed FIESC haplotype; green box indicates unresolved Trinidad Equiseti sequences.

The three-locus dataset consisted of concatenated *EF-1α*, *CAM*, and *RPB2* partial gene sequences based on their demonstrated phylogenetic informativeness within the genus (GenBank Accession Nos. MN729351 to MN729362). Reference sequence data used to construct phylogenetic trees is detailed in Table 1. Phylogenetic analyses identified three different MLST haplotypes of *F. incarnatum* indicative of three phylogenetic species and one MLST group of *F. equiseti* indicative of just one phylogenetic species. The *F. incarnatum* membership species were FIESC-15a, FIESC-16a, and FIESC-26a. The *F. equiseti* membership species could not be determined as the Trinidad isolates clustered separately with high bootstrap support from all other Equiseti species, but was confirmed to be a member species of the Equiseti clade. These four Trinidad *F. equiseti* isolates represented 8% of the total number of isolates that were subjected to genetic typing. The remaining Trinidad isolates belonged to the Incarnatum clade as phylogenetic species FIESC-15a (22%), FIESC-16a (48%), and FIESC-26a (22%) and these sequences were resolved for each individual locus. Bootstrapping of the three-gene concatenated dataset provided strong support for several distinct relationships among the MLST haplotypes of FIESC and enabled the identification of *F. equiseti* species that, phylogenetically, may be new to the already known haplotypes (Figure 1).

### 2.2. Tebuconazole Phenotypes 

Representative isolates from each group were selected for fungicide screens: *F. incarnatum*: FIESC-15a (N = 9); FIESC-16a (N = 21); FIESC-26a (N = 12) and *F. equiseti* (N = 4) (Table 2; Table 3). EC50 values were significantly different among the MLST groups (*p* ≤ 0.001). Tukey (HSD) and LSD (T) comparisons of EC50 values revealed two homogeneous groups: one group consisted of isolates belonging to *F. equiseti* and FIESC-26a, and the other group consisted of isolates belonging to FIESC-15a and FIESC-16a. The EC50 values of isolates between these two groups were significantly different (*p* ≤ 0.01); however, there were no significant differences in EC50 values within *F. equiseti* and FIESC-26a, *p* = 0.6879; and within FIESC-15a and FIESC-16a, *p* = 0.4059 (Supplementary data file 1: Statistical analysis—Tables S1 and S2 (a–e)). FIESC isolates demonstrated one of two phenotypes: “less sensitive” to 10 µg/mL tebuconazole (isolates belonging to FIESC-15a and FIESC-16a), and “more sensitive” to 10 µg/mL tebuconazole (FIESC-26a and *F. equiseti*). 

### 2.3. CYP51C Sequence Analysis 

Analysis of the conservation plot of the aligned FIESC CYP51C nucleotide sequences from the Trinidad isolates revealed five polymorphic sites: nt position 51 C > T; nt position 69 A > G; nt position 221 T > G; nt position 423 A > G; nt position 647 C > T. All other sites were highly conserved for all isolates included in the dataset. Haplotype analysis revealed five CYP51C haplotypes (h = 5). Haplotype 1 was shared by twenty-seven isolates; haplotype 2 was shared by two isolates, 31 and 36; haplotype 3 was shared by the nine isolates of the second cluster; haplotype 4 was shared by two isolates, 38 and 49; haplotype 5 was shared by two isolates 13 and 14. Haplotype diversity (Hd) for the aligned CYP51C gene sequences was 0.540, and low estimates are <0.5. There were no recombination footprints for the FIESC CYP51C sequences of the Trinidad isolates based on Hudson’s r estimation of the recombination rate per sequence or per site in DnaSP. RDP3 also did not detect any signatures of recombination. The nonsynonymous/synonymous substitution sites ratio, dN/dS ratio, was >1 (value = 3.46), which suggested that the sequences were under positive selection [52]. CYP51C nucleotide and deduced amino acid sequence had no correlation to azole sensitivity in this study. 

**Figure 2:**
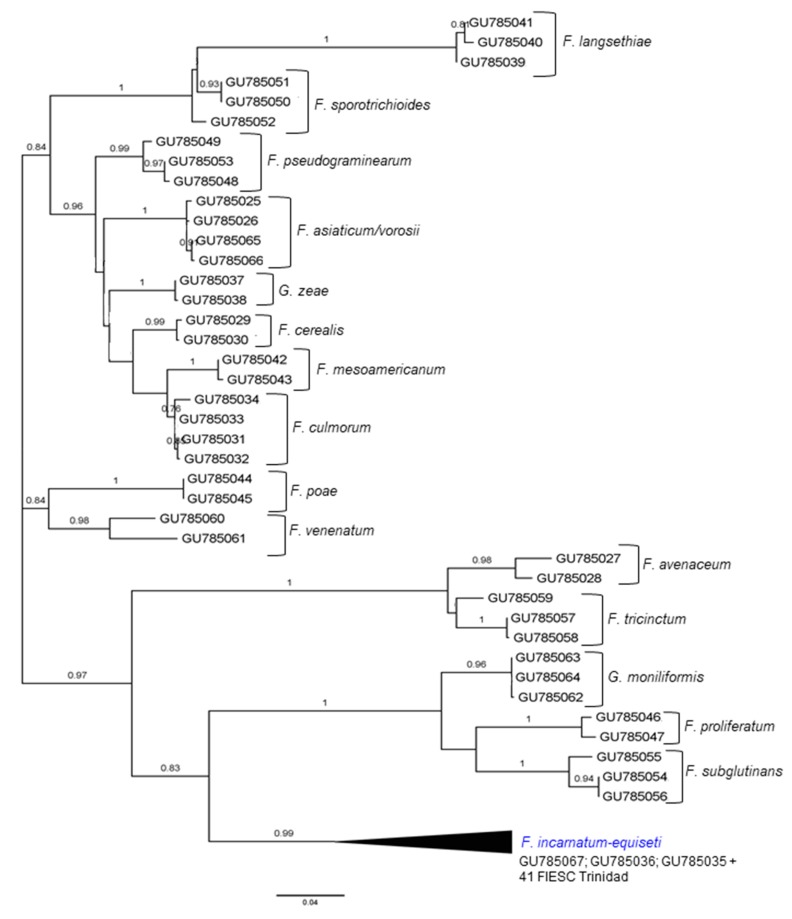
Phylogenetic analysis of CYP51C partial gene sequences of FIESC isolates from Trinidad and other geographical regions by Maximum Likelihood method based on the General Time Reversible model, as the best fit model, with 1000 bootstrapped replicates [50]. The tree with the highest log-likelihood is shown. The tree is drawn to scale, with branch lengths measured in the number of substitutions per site. The analysis involved 81 nucleotide sequences. All positions containing gaps and missing data were eliminated. There was a total of 259 positions in the final dataset. Evolutionary analyses were conducted in MEGA6 [51].

Phylogenetic analysis of CYP51C sequences was carried out on a final sequence dataset of 81 nucleotide sequences. Phylogenetic inference revealed polytomic branching for 41 FIESC Trinidad isolates with the other reference FIESC sequences, which is illustrated as a collapsed branch in the ML tree. The majority of reference *Fusarium* sequences were resolved into distinct, species-specific clusters (Figure 2), indicating that CYP51C sequences may have species-specific signatures that are absent among members of FIESC as a species complex and explains why FIESC sequences could not be resolved.

## 3. Discussion

Members of FIESC from Trinidad were identified to the phylogenetic species level but only for the Incarnatum clade membership. Three Incarnatum species, FIESC-15a, FIESC-16a, and FIESC-26a, and one Equiseti species, were identified based on a three-locus sequence comparison scheme. *EF-1*α, *RPB2*, and *CAM* markers were successful in resolving the Trinidad *F. incarnatum* isolates. Other studies reported that the *CAM* and *RPB2* gene sequences were more successful at identifying isolates within the FCSC and FIESC associated with human and animal infections [14,53]. Conversely, it was found that the *RPB2* locus was less discriminatory than *EF-1*α sequences in a study of 25 clinical isolates in China [54]. The results of the three-locus DNA typing scheme discussed herein extend and provide additional data on the species/species complex distribution and genetic diversity of major pathogenic FIESC isolates, which was previously based on one locus (*EF-1*α) as the ITS sequences were not phylogenetically informative [8]. While partial sequences of *EF-1*α have proven to be extraordinarily useful for resolving species boundaries in *Fusarium* [18,55,56,57,58], most of the intronic sequences are too divergent to align beyond the species complex. For this reason, *RPB1* and *RPB2* nucleotide sequences are more informative for genus-wide phylogenetics within *Fusarium*. However, in this study, *CAM* and *RPB2* nucleotide sequences were unable to assign Trinidad Equiseti isolates to MLST haplotype within the Equiseti clade. Additional loci of *GAPDH* and *ACT* nucleotide sequences were also unable to resolve Trinidad Equiseti isolates to MLST haplotype (data not shown).

To date, considerable effort has been expended to devise accurate approaches to molecular phylogenetic analysis of *Fusarium* species. Depending on the species and the species complex, different combinations of markers whereby the locus and the number of markers must enable species-specific sequence identification [53]. A single-locus (*EF-1*α gene) best match at <99.4% sequence identity indicates that this query species may not be represented in the database for this locus and, therefore, sequence data from additional loci are recommended to identify phylogenetic species accurately [53]. Two loci, *EF-1*α and RBP2, allow species identification within the *Gibberella* (*Fusarium fujikuroi*) species complex GFSC, but for *F. oxysporum* species complex (FOSC), the loci recommended are *EF-1*α and IGS [14]. To discriminate among cryptic species within the *Fusarium solani* species complex (FSSC), FIESC, and *F. chlamydosporum* species complex (FCSC), ≥four loci are recommended: *EF-1*α, *CAM*, *RPB2*, ITS [53].

Generally, species delimitation within the FIESC is still poorly defined. Within the *Fusarium incarnatum-equiseti* species complex (FIESC) and using multiple loci, at least 33 species can be recognized, which are organized into two main clades: MLST haplotypes 1–14 are molecular siblings of *F. equiseti*, while the remaining MLST haplotypes are grouped as ‘*F. incarnatum*’ [14]. Species assignment within the Equiseti clade, according to Wang et al. [59] includes: *F. ipomoeae*-FIESC-1; *F. compactum*-FIESC-3; *F. equiseti*, *F. incarnatum*, *F. lacertarum*-FIESC-4; *F. arcuatisporum*-FIESC-7; *F. scirpi*-FIESC-9; and *F. equiseti*-FIESC-14. The Incarnatum clade consists of: *F. irregulare*-FIESC-15; *F. sulawense*-FIESC-16/-17; *F. luffe*-FIESC-18; *F. guillinense*-FIESC-21; *F. nanum*-FIESC-25; *F. hainanense*-FIESC-26; *F. citri*-FIESC-29; and *F. humuli*-FIESC-33. However, these species may not be universally accepted in all indexes, for example, *F. scirpi* is currently listed as a synonym of *F. acuminatum* in the Index Fungorum (http://www.indexfungorum.org/), but is a distinct species in MycoBank (http://www.mycobank.org/).

Of the Incarnatum haplotypes detected in Trinidad, FIESC-15, identified as *F. irregulare*, is commonly associated with human infections in the USA; FIESC-16, identified as *F. sulawense*, is associated with both human and plant diseases including *Capsicum* species. FIESC-26, identified as *F. hainanense*, is associated with only plant diseases to date [59]. Observed fusarioses range from onychomycoses, skin infections, and keratitis, mainly in healthy individuals, to deep local and disseminated infections in immunocompromised, predominantly in leukemia patients with a high mortality rate. In general, like many other members of the order Hypocreales, *Fusarium* species are highly refractory to antifungal therapy. To reveal small differences in susceptibility between clinically relevant *Fusarium* species, precise identification of isolates is recommended [60]. This suggests that the detection of FIESC-15 and FIESC-16 Trinidad isolates may have clinical implications.

Since their introduction to agriculture over three decades ago, reduced sensitivity to azoles has been reported for several important phytopathogenic fungi, including *F. graminearum* [33,61], *Erysiphe graminis* [62], *Monilinia fructicola* [63], and *Mycosphaerella graminicola* [64]. Three molecular mechanisms have been described that may explain a “less sensitive” or “less resistant” phenotype against azole fungicides [34,65,66,67,68,69]. The azoles used in agriculture and in clinical settings target the same active site, which means that pathogenic fungi can engage shared modes of resistance [70]. Faria-Ramos et al. [71] and Berger et al. [72] reported the resistance of *Aspergillus* sp. to clinically relevant azole fungicides was due to exposure to agricultural azoles which led to the emergence of cross-resistance. 

Our findings indicated that, for field isolates of FIESC in Trinidad, there was an association between specific phylogenetic species and tebuconazole sensitivity. FIESC-26a and *F. equiseti* species were sensitive to 10 µg/mL of tebuconazole fungicide as a discriminatory dose in in vitro bioassays. In this study, there was no correlation between tebuconazole sensitivity and CYP51C haplotypes. A study of *Rhynchosporium commune* isolates in the UK revealed similar findings where the CYP51B gene: (i) was under positive selection, (ii) had no signatures of recombination, (iii) had little nucleotide diversity, and (iv) neither amino acid sequence nor haplotypes were associated with azole sensitivity [73]. It is proposed that in such cases, the paralogue acquired and retained a function that was different from the ancestral type but was one that enabled evolutionary adaptation and survival of pathogenic fungi.

The CYP51C gene was present in all FIESC isolates included in this study; however, while five polymorphic sites were found in the aligned nucleotide sequences of the Trinidad isolates, there was absolute amino acid conservation. This low level of diversity, together with evidence of positive selection for the CYP51C gene and a polytomic phylogenetic relationship, indicate that CYP51C is a conserved functional paralogue among these FIESC isolates and it is under selective constraints. Not all CYP51 gene duplications result in functional conservation of the copied gene, for example, Hawkins et al. [74] reported the existence of two paralogues of CYP51 gene in *R. commune*: CYP51A, CYP51B and a duplicated copy of CYP51A considered to be a pseudogene (CYP51A-p) because of its high nucleotide sequence diversity, it was not under purifying selection and was not functional. Paralogues tend to persist in a given genome when one of two paralogues undergo positive selection due to gain a novel function (neofunctionalization), while the other paralogue preserves the ancestral function or where the paralogues partition the ancestral function [75,76,77].

There are increasing reports of plant pathogenic *Fusarium* species implicated in opportunistic and systemic infections in humans and animals, which suggests that these Fusaria are able to engage pathogenic strategies to infect plants as well as animals and humans [78]. This trans-kingdom pathogenicity may be due to a number of virulence factors which perhaps include CYP51C. 

## 4. Materials and Methods 

### 4.1. Collection of Isolates 

Bell pepper fields located in the main growing areas in Trinidad were visited: Aranguez (north and south), Orange Grove, Maloney, Caura, Caroni, Bon aventure, Penal, Mayo. Red bell pepper fruits showing typical symptoms of FIESC infection were collected in plastic bags and were transported to the lab. Symptoms of infection included large watery lesions that expanded to the calyx end of the fruit with internal rot of the seed placenta in severely infected fruit. The fruits were surface sterilized by rinsing in 70% ethanol for 1 min followed by another rinse in 0.6% sodium hypochlorite solution for 1 min. Samples were then washed three times in sterilized distilled water and air-dried. Blocks of fruit tissue (5 mm^3^) were removed from the margins of the lesions and transferred to potato dextrose agar (PDA) media (Oxoid Ltd., UK) supplemented with 50 mg/L streptomycin, tetracycline, and chloramphenicol. Plates were incubated for seven days in the dark at 25 °C. Monoconidial cultures were subsequently obtained and maintained on PDA at 4 °C for temporary storage, and as conidial suspensions in 50% glycerol at −70 °C for long-term storage. The number of isolates according to field location were as follows: Aranguez (N = 12); Macoya (N = 27); Maloney (N = 19); Central (N = 6); Penal (N = 5); Bon Aventure (N = 10).

### 4.2. DNA Extraction, PCR Amplification, and Sequencing 

DNA was extracted from actively growing colonies using the Maxwell^®^-16 automated DNA extraction system (Promega, Madison, Wisconsin, USA) based on magnetic bead capture DNA extraction according to the manufacturer’s instructions. The *EF-1*α gene of 50 isolates in the Trinidad collection was amplified using published protocols [14,55,79]. PCR products were sequenced directly (MCLAB, San Francisco, USA). Nucleotide sequences were aligned using MAFFT (Multiple Alignment using Fast Fourier Transform) alignment programs (https://www.ebi.ac.uk/Tools/msa/mafft/; [80]). Sequences were then edited using BioEdit sequence alignment editor software version 7.2.5 (http://www.mbio.ncsu.edu/bioedit/page2.html). A homology search was carried out for the *EF-1*α sequences in *Fusarium* MLST [14,53].

### 4.3. Multi-locus Sequencing Typing (MLST) for Phylogenetic Species Identification 

Species designations were based on the multi-locus haplotype system of O’ Donnell et al. [14]. Arabic numerals were used to assign isolates to phylogenetic species, and lowercase Roman letters were used to indicate a >99.4% sequence match to the unique haplotype in the *Fusarium* MLST database. Partial sequences of three gene regions were used: *EF-1α* (598 bp) [79], *RPB2* (primers: *RPB2*-5f2 and *RPB2*-7cr, amplicon size 1750 bp) [53] and *CAM* (primers CL1 and CL2, amplicon size 700 bp) [81]. Thermal cycling conditions for amplifying *EF-1α* and *RPB2* were carried out as described by O’Donnell et al. [53,79]. Amplification of the *CAM* gene region was as described by Cai et al. [81] and Prihastuti et al. [82]. The specific gene regions and the number of loci used for MLST were determined according to the study by O’Donnell et al. [14]. A three-locus scheme allowed for more robust genetic typing. Twenty representative isolates were used in sequence comparisons of *RPB2* and *CAM* gene regions based on the homology search to identify *EF-1*α sequences in *Fusarium* MLST. Two additional loci were amplified according to the PCR conditions described by Prihastuti et al. [82], Actin (*ACT*; Primers: ACT512F 5′-ATGTGCAAGGCCGGTTTCGC-3′ and ACT783R 5′-TACGAGTCCTTCTGGCCCAT-3′) [82] and Glyceradehyde-3-phosphate dehydrogenase (*GAPDH*; Primers: GAPDHF1 5′-GCCGTCAACGACCCCTTCATTGA3′ and GDR1 5′-GGGTGGAGTCGTACTTGAGCATGT-3′) [82] and the amplicons were sequenced. However, these sequences are not curated in the CBS-KNAW culture collection, and BLASTn searches in GenBank indicated assignment only to the genus level and not to FIESC phylogenetic species level. Therefore, these *ACT* and *GAPDH* sequences were omitted from the final MLST scheme. 

### 4.4. Phylogenetic Analysis 

For each locus, sequences were aligned using the MAFFT v. 7 (Multiple Alignment using Fast Fourier Transform) [80], and the alignments were manually adjusted in BioEdit [83]. Phylogenetic relationships of both individual gene and concatenated gene datasets were inferred by the maximum likelihood (ML) algorithm using MEGA6 (https://www.megasoftware.net/) [51] and PhyML v.3.0 (http://www.atgc-montpellier.fr/phyml/) [84] software. The best fit model of nucleotide substitution for each locus was determined in MEGA6 by examining the Bayesian and Akaike criterion information scores, as well as the log-likelihood scores. Kimura-2-Parameter (K-2-P+G/I) was found to be the simplest best fit model for each locus. A more complex model was also applied (GTR+G+I) for each locus to compare branching, taxon placement, and bootstrap scores in phylogenetic trees generated with the K-2-P+G model. Non-uniformity of evolutionary rates among sites were compensated for by using a discrete Gamma distribution (+G) with five rate categories with the assumption that a proportion of sites are evolutionarily invariable (+I). The parameters were the same for both models, and therefore, the GTR+G+I model was used for the concatenated data. Bootstrap values over 75% were considered significant and, therefore, the rooted, 75% consensus tree is presented. Sequences of the *EF-1*α, *RPB2* and *CAM* datasets of O’Donnell et al. [14] were used in phylogenetic analyses: FIESC *EF-1*α PopSet: 262476356, FIESC *RPB2* PopSet: 262476623, FIESC *CAM* PopSet: 262476268. 

### 4.5. Fungicide Sensitivity 

The sensitivity of FIESC isolates to tebuconazole was assessed in an in vitro radial mycelial growth assay. The PDA media were amended with 0.0, 0.1, 1.0, 10.0, and 100.0 μg/mL of a commercial formulation of the fungicide (“Tebizole-25% WP”-Veterinary and agricultural products manufacturing company Ltd., Jordan, Israel). Stock solutions of the fungicide were prepared in acetone, and an acetone-only control was included [85]. The commercial fungicide preparation was insoluble in sterile distilled water. Four replicates of each fungicide concentration were used for each isolate, and the experiment was performed twice. Blocks (4-mm^3^) were moved from the advancing edge of actively growing colonies and placed, mycelium-side down, in the center of fungicide amended medium. The plates were incubated at 25 °C for five days, and the radial diameter of each colony was measured (orthogonal measurements) for each isolate to determine the percentage of relative growth inhibited compared to the growth on non-amended media. The measurement data for both replicates did not differ significantly based on Fisher’s Least Significant Difference Test (LSD) at *p* ≤ 0.05. Mean diameter values were, therefore, used in subsequent analyses. Linear regression analysis of the percentage of growth inhibition (mycelia growth of the control versus the log10 of the fungicide concentration) was carried out in MINITAB v.17 (State College, PA, USA). The effective concentration required to achieve 50% colony growth inhibition (EC50) on fungicide-amended media was calculated for isolates according to MLST. The DMI fungicides have no effect on spore germination and early germ tube growth because of sterol reserves in spores [86]; therefore, the effect of tebuconazole on spore germination was not investigated here.

### 4.6. Genetic Structure of CYP51C

CYP51C sequences were amplified by primers designed by Fernández-Ortuño et al. [87]. Sequencing was done as described in the previous section. Nucleotide sequences were aligned with the MAFFT alignment program with manual sequence adjustments in BioEdit. The final alignment consisted of 81 CYP51C sequences: 41 sequences belonged to FIESC Trinidad, and the other 40 belonged to PopSet: 292660854 [87].

The relative degree of DNA polymorphism, nucleotide divergence, and haplotype analysis were determined for CYP51C nucleotide sequences using DnaSP (DNA Sequence Polymorphism software version 5.10) [88,89]. DnaSP software was also used to determine whether CYP51C sequences were under positive selection and to find evidence of recombination. RDP3 software was used for characterizing recombination events, visualizing patterns of recombination, and recombination-aware ancestral sequence reconstruction. The CYP51C nucleotide sequences were translated using the EMBOSS Transeq software (https://www.ebi.ac.uk/Tools/st/emboss_transeq/) and aligned using Clustal Omega (https://www.ebi.ac.uk/Tools/msa/clustalo/). The amino acid alignment was edited, and the conservation plot was analyzed in BioEdit. Phylogenetic analysis was carried out as previously described.

## Figures and Tables

**Table 1:** Reference sequences used in the phylogenetic study.

*CAM*	*EF1a*	*RPB2*	NRRL	FIESC Haplotype	Host	Country	Reference
GQ505575	GQ505664	GQ505482	43637	1-a	dog	Pennsylvania	[14]
GQ505582	GQ505671	GQ505849	45996	1-a	human sinus	New York	[14]
GQ505578	GQ505667	GQ505845	43640	1-a	dog nose	Texas	[14]
GQ505551	GQ505639	GQ505817	34039	1-b	human	Connecticut	[14]
GQ505548	GQ505636	GQ505814	34034	1-c	human leg	Arizona	[14]
GQ505563	GQ505651	GQ505829	36401	2-a	cotton	Mozambique	[14]
GQ505564	GQ505652	GQ505830	36448	2-b	*Phaseolus vulgaris* seed	Sudan	[14]
GQ505824	GQ505646	GQ505558	36318	3-a	unknown	unknown	[14]
GQ505560	GQ505648	GQ505826	36323	3-a	cotton yarn	England	[14]
GQ505514	GQ505602	GQ505780	28029	3-b	Human eye	California	[14]
GQ505505	GQ505593	GQ505771	20423	4-a	Lizard skin	India	[14]
GQ505555	GQ505643	GQ505821	36123	4-b	unknown	unknown	[14]
GQ505531	GQ505619	GQ505797	32871	5-a	human abscess	Texas	[14]
GQ505547	GQ505635	GQ505813	34032	5-a	human abscess	Texas	[14]
GQ505550	GQ505638	GQ505816	34037	5-b	human abscess	Colorado	[14]
GQ505581	GQ505670	GQ505848	45995	5-b	human abscess	Colorado	[14]
GQ505509	GQ505597	GQ505775	25795	5-c	*Disphyma* seed	Germany	[14]
GQ505549	GQ505637	GQ505815	34035	5-d	human sinus	Colorado	[14]
GQ505572	GQ505661	GQ505839	43623	5-e	human maxillary sinus	Colorado	[14]
GQ505583	GQ505672	GQ505850	45997	5-f	human sinus	Colorado	[14]
GQ505576	GQ505665	GQ505843	43638	6-a	Manatee	Florida	[14]
GQ505579	GQ505668	GQ505846	43694	6-a	human eye	Texas	[14]
GQ505584	GQ505673	GQ505851	45998	6-b	human toe	Texas	[14]
GQ505536	GQ505642	GQ505802	32997	7-a	human toe nail	Colorado	[14]
GQ505500	GQ505588	GQ505766	5537	8-a	Fescue hay	Missouri	[14]
N/A	GQ505658	GQ505836	43498	8-b	human eye	Pennsylvania	[14]
GQ505566	GQ505654	GQ505832	36478	9-a	Pasture soil	Australia	[14]
GQ505517	GQ505604	GQ505783	29134	9-a	Pasture soil	Australia	[14]
GQ505504	GQ505592	GQ505770	13402	9-b	Pine soil	Australia	[14]
GQ505513	GQ505601	GQ505779	26922	9-c	soil	France	[14]
GQ505498	GQ505586	GQ505764	3020	10-a	unknown	unknown	[14]
GQ505499	GQ505587	GQ505765	3214	10-a	unknown	unknown	[14]
GQ505561	GQ505649	GQ505827	36372	11-a	air	Netherlands	[14]
GQ505501	GQ505589	GQ505767	6548	12-a	Wheat	Germany	[14]
GQ505512	GQ505600	GQ505778	26921	12-a	Wheat	Germany	[14]
GQ505518	GQ505606	GQ505784	31011	12-a	*Thuja* sp.	Germany	[14]
GQ505557	GQ505645	GQ505823	36269	12-b	*Pinusnigra* seedling	Croatia	[14]
GQ505562	GQ505650	GQ505828	36392	12-c	seedling	Germany	[14]
GQ505573	GQ505662	GQ505840	43635	13-a	horse	Nebraska	[14]
GQ505511	GQ505599	GQ505777	26419	14-a	soil	Germany	[14]
GQ505556	GQ505644	GQ505822	36136	14-a	unknown	unknown	[14]
GQ505559	GQ505647	GQ505825	36321	14-a	soil	Netherlands	[14]
GQ595565	GQ505653	GQ505831	36466	14-a	potato peel	Denmark	[14]
GQ505506	GQ505594	GQ505772	20697	14-b	beet	Chile	[14]
GQ505574	GQ505663	GQ505841	43636	14-c	dog	Texas	[14]
GQ505521	GQ505609	GQ505787	32175	15-a	human septum	Texas	[14]
GQ505542	GQ505630	GQ505808	34006	15-a	human eye	Texas	[14]
GQ505543	GQ505631	GQ505809	34007	15-a	human septum	Texas	[14]
GQ505546	GQ505634	GQ505812	34011	15-a	human septum	Texas	[14]
GQ505570	GQ505659	GQ505837	43619	15-a	human finger	Texas	[14]
GQ505523	GQ505611	GQ505789	32182	15-b	human blood	Texas	[14]
GQ505519	GQ505607	GQ505785	31160	15-c	human lung	Texas	[14]
GQ505522	GQ505610	GQ505788	32181	15-c	human blood	Oklahoma	[14]
GQ505530	GQ505618	GQ505796	32869	15-c	human cancer patient	Texas	[14]
GQ505533	GQ505621	GQ505799	32994	15-c	human ethmoid sinus	Texas	[14]
GQ505534	GQ505622	GQ505800	32995	15-c	human sinus	Texas	[14]
GQ505535	GQ505623	GQ505801	32996	15-c	human leg wound	Texas	[14]
GQ505545	GQ505633	GQ505811	34010	15-c	human maxillary sinus	Texas	[14]
GQ505571	GQ505660	GQ505838	43622	15-c	human lung	Texas	[14]
GQ505544	GQ505632	GQ505810	34008	15-d	human lung	Texas	[14]
GQ505537	GQ505625	GQ505803	34001	15-e	human foot wound	Texas	[14]
GQ505540	GQ505628	GQ505806	34004	16-a	human BAL	Texas	[14]
GQ505552	GQ505640	GQ505818	34056	16-b	human bronchial wash	Illinois	[14]
GQ505553	GQ505641	GQ505819	34059	16-c	human blood	Illinois	[14]
GQ505580	GQ505669	GQ505847	43730	16-c	Contact lens	Mississippi	[14]
GQ505525	GQ505613	GQ505791	32864	17-a	human	Texas	[14]
GQ505567	GQ505655	GQ505833	36548	17-b	Banana	Congo	[14]
GQ505554	GQ505642	GQ505820	34070	17-c	Tortoise	Illinois	[14]
GQ505520	GQ505608	GQ505786	31167	18-a	human septum	Texas	[14]
GQ505524	GQ505612	GQ505790	32522	18-b	human diabetic cellulitis	Illinois	[14]
GQ505577	GQ505666	GQ505844	43639	19-a	Manatee	Florida	[14]
GQ505539	GQ505627	GQ505805	34003	20-a	human septum	Texas	[14]
GQ505568	GQ505656	GQ505834	36575	20-b	*Juniperus chinensis* leaf	Hawaii	[14]
GQ505502	GQ505590	GQ505768	13335	21-a	alfalfa	Australia	[14]
GQ505526	GQ505614	GQ505792	32865	21-b	human endocarditis	Brazil	[14]
GQ505538	GQ505626	GQ505804	34002	22-a	human ethmoid sinus	Texas	[14]
GQ505527	GQ505615	GQ505793	32866	23-a	human cancer patient	Texas	[14]
GQ505528	GQ505618	GQ505794	32867	23-a	human	Texas	[14]
GQ505503	GQ505591	GQ505769	13379	23-b	*Oryza sativa*	India	[14]
GQ505541	GQ505629	GQ505807	34005	24-a	human intravitral fluid	Minnesota	[14]
GQ505569	GQ505657	GQ505835	43297	24-b	*Saprotina* rhizomes	Connecticut	[14]
GQ505508	GQ505596	GQ505774	22244	25-a	rice	China	[14]
GQ505532	GQ505620	GQ505798	32993	25-b	human nasal tissue	Texas	[14]
GQ505529	GQ505617	GQ505795	32868	25-c	human blood	Texas	[14]
GQ505510	GQ505598	GQ505776	26417	26-a	leaf litter	Cuba	[14]
GQ505516	GQ505604	GQ505782	28714	26-b	*Acacia* sp. Branch	Costa Rica	[14]
GQ505507	GQ505595	GQ505773	20722	27-a	*Chrysanthemum* sp.	Kenya	[14]
GQ505515	GQ505603	GQ505781	28577	28-a	grave stone	Romania	[14]
GQ505585	GQ505674	GQ505852	13459	N/A	plant debris	South Africa	[14]

**Table 2:** EC50 data for isolates belonging to FIESC-15a and FIESC-16a.

Sample	MLST Type	Growth Inhibition (%) ^1^	EC50 (µg/mL)
3	15-a	52.94	9.1
13	15-a	49.02	10.4
15	15-a	54.90	7.8
20	15-a	60.61	5.5
22	15-a	52.38	9.5
28	15-a	43.48	13.3
31	15-a	55.56	7.7
33	15-a	62.86	4.7
36	15-a	52.94	9.1
2	16-a	48.39	10.1
5	16-a	42.42	13.8
6	16-a	56.41	7.2
14	16-a	58.82	6.3
18	16-a	39.39	10.8
21	16-a	64.29	4.3
25	16-a	64.29	4.3
26	16-a	58.82	6.4
27	16-a	62.86	4.7
29	16-a	31.58	18.4
32	16-a	35.00	17.1
35	16-a	48.39	10.9
38	16-a	42.42	13.8
39	16-a	56.41	7.7
40	16-a	52.94	8.7
41	16-a	49.02	10.5
42	16-a	50.98	11.5
47	16-a	39.39	10.8
49	16-a	45.45	5.5
50	16-a	53.85	12.4
51	16-a	49.02	10.5

^1^ Growth inhibition was determined for 10 µg/mL of tebuconazole.

**Table 3:** EC50 data for isolates belonging to FIESC-26a and *Fusarium equiseti*.

Sample	MLST Type	Growth Inhibition (%) ^1^	EC50 (µg/mL)
56	26-a	80.00	1.2
57	26-a	82.05	1.2
58	26-a	80.00	1.2
59	26-a	80.56	1.5
60	26-a	81.58	2.3
61	26-a	80.56	5.8
62	26-a	80.00	1.9
63	26-a	80.00	1.3
64	26-a	80.00	2.7
65	26-a	80.00	3.2
66	26-a	80.56	1.9
67	26-a	80.00	1.6
52	Equiseti	100.00	2.6
53	Equiseti	100.00	2.6
54	Equiseti	100.00	3.6
55	Equiseti	100.00	1.8

^1^ Growth inhibition was determined for 10 µg/mL of tebuconazole.

## Supplementary Materials

The following are available online at https://www.mdpi.com/2076-0817/9/3/175/s1, Table S1: Summary statistics for percentage growth inhibition; Table S2: Summary statistics for EC50 data.
