# Supplementary material for: Three-Locus Sequence Identification and Differential Tebuconazole Sensitivity Suggest Novel Fusarium equiseti Haplotype from Trinidad
Source: Pathogens. 2020 Mar 1;9(3):175. doi: 10.3390/pathogens9030175 (PMC7157627; doi:10.3390/pathogens9030175)
Supplement: Supplementary file 1 [file pathogens-09-00175-s001.pdf]

Supplementary data.

Table S1 (a). Summary statistics for percentage growth inhibition data for each MLST group

	MLST 11	MLST 15	MLST 16	MLST 26
N	4	6	29	17
MEAN	100.00	59.51	50.85	80.23
SD	0.00	3.10	18.17	6.95
SE MEAN	0.00	1.27	3.37	1.69
MINIMUM	100.00	55.00	3.85	66.07
MAXIMUM	100.00	61.91	74.58	100.00

SD – Standard deviation

SE – Standard error of the mean

Table S1 (b). One-way ANOVA for arcsine transformed % growth inhibition for all MLST groups

SOURCE	DF	SS	MS	F	*P*
BETWEEN	3	4.696	1.565	48.630	0.000
WITHIN	52	1.674	0.032		
TOTAL	55	6.370			

DF – Degrees of freedom

SS – Sum of squares

MS – Mean sum of squares

F – *F* statistic

P – Significance level at 0.05

Table S1 (c). One-way ANOVA for arcsine transformed % growth inhibition for MLST 11 and MLST 15

SOURCE	DF	SS	MS	F	*P*
BETWEEN	1	2.089	2.089	2282.320	0.000
WITHIN	8	0.007	0.001		
TOTAL	9	2.096			

DF – Degrees of freedom

SS – Sum of squares

MS – Mean sum of squares

F – *F* statistic

P – Significance level at 0.05

Table S1 (d). One-way ANOVA for arcsine transformed % growth inhibition for MLST 11 and MLST 16

SOURCE	DF	SS	MS	F	*P*
BETWEEN	1	3.704	3.704	97.740	0.000
WITHIN	31	1.175	0.038		
TOTAL	32	4.879			

DF – Degrees of freedom

SS – Sum of squares

MS – Mean sum of squares

F – *F* statistic

P – Significance level at 0.05

Table S1 (e). One-way ANOVA for arcsine transformed % growth inhibition for MLST 11 and MLST 26

SOURCE	DF	SS	MS	F	*P*
BETWEEN	1	1.237	1.237	47.800	0.000
WITHIN	19	0.492	0.026		
TOTAL	20	1.729			

DF – Degrees of freedom

SS – Sum of squares

MS – Mean sum of squares

F – *F* statistic

P – Significance level at 0.05

Table S2 (a). Summary statistics for EC50 data for each MLST group

	MLST 11	MLST 15	MLST 16	MLST 26
N	4	9	21	12
MEAN	1.87	8.57	9.79	2.15
SD	0.06	2.56	3.97	1.31
SE MEAN	0.03	0.85	0.87	0.38
MINIMUM	1.80	4.74	4.25	1.20
MAXIMUM	1.94	13.30	18.39	5.77

SD – Standard deviation

SE – Standard error of the mean

Table S2 (b). Analysis of variance (ANOVA) of EC50 values for all MLST groups

	DF	SS	MS	F	*P*
BETWEEN	3	572.39	190.80	20.75	0.000
WITHIN	42	386.11	9.19		
TOTAL	45	958.50			

DF – Degrees of freedom

SS – Sum of squares

MS – Mean sum of squares

F – *F* statistic

P – Significance level at 0.05

Table S2 (c). Tukey (HSD) comparison of mean EC50 values indicates where the EC50 values are significantly different according to MLST group

VARIABLE	MEAN	GROUPS[1]
MLST 16	9.7876	I
MLST 15	8.57	I
MLST 26	2.1467	.. I
MLST 11	1.8725	.. I

[1] – LSD (T) comparison of mean EC50 values were identical to Tukey test.

Table S2 (d). Analysis of variance (ANOVA) of EC50 values for MLST 11 and MLST 26

	DF	SS	MS	F	*P*
BETWEEN	1	0.2255	0.2255	0.17	0.6879
WITHIN	14	18.7691	1.34065		
TOTAL	15	18.9946			

DF – Degrees of freedom

SS – Sum of squares

MS – Mean sum of squares

F – *F* statistic

P – Significance level at 0.05

Table S2 (e). Analysis of variance (ANOVA) of EC50 values for MLST 15 and MLST 16

	DF	SS	MS	F	*P*
BETWEEN	1	9.34036	9.34036	0.71	0.4059
WITHIN	28	367.34	13.1193		
TOTAL	29	376.68			

DF – Degrees of freedom

SS – Sum of squares

MS – Mean sum of squares

F – *F* statistic

P – Significance level at 0.05
